# Release of free amino acids upon oxidation of peptides and proteins by hydroxyl radicals

**DOI:** 10.1007/s00216-017-0188-y

**Published:** 2017-01-20

**Authors:** Fobang Liu, Senchao Lai, Haijie Tong, Pascale S. J. Lakey, Manabu Shiraiwa, Michael G. Weller, Ulrich Pöschl, Christopher J. Kampf

**Affiliations:** 1 Multiphase Chemistry Department, Max Planck Institute for Chemistry, Hahn-Meitner-Weg 1, 55128 Mainz, Germany; 2 School of Environment and Energy, South China University of Technology, Higher Education Mega Center, Guangzhou, 510006 China; 3 Department of Chemistry, University of California, Irvine, 1102 Natural Sciences II, Irvine, CA 92697-2025 USA; 4 Division 1.5 Protein Analysis, Federal Institute for Materials Research and Testing (BAM), Richard-Willstätter-Str. 11, 12489 Berlin, Germany; 5 Institute for Inorganic and Analytical Chemistry, Johannes Gutenberg University Mainz, Duesbergweg 10-14, 55128 Mainz, Germany; 6 Institute for Organic Chemistry, Johannes Gutenberg University Mainz, Duesbergweg 10-14, 55128 Mainz, Germany

**Keywords:** Peptides, Proteins, Oxidation, Hydroxyl radicals, HPLC-MS, Amino acid analysis

## Abstract

Hydroxyl radical-induced oxidation of proteins and peptides can lead to the cleavage of the peptide, leading to a release of fragments. Here, we used high-performance liquid chromatography tandem mass spectrometry (HPLC-MS/MS) and pre-column online *ortho*-phthalaldehyde (OPA) derivatization-based amino acid analysis by HPLC with diode array detection and fluorescence detection to identify and quantify free amino acids released upon oxidation of proteins and peptides by hydroxyl radicals. Bovine serum albumin (BSA), ovalbumin (OVA) as model proteins, and synthetic tripeptides (comprised of varying compositions of the amino acids Gly, Ala, Ser, and Met) were used for reactions with hydroxyl radicals, which were generated by the Fenton reaction of iron ions and hydrogen peroxide. The molar yields of free glycine, aspartic acid, asparagine, and alanine per peptide or protein varied between 4 and 55%. For protein oxidation reactions, the molar yields of Gly (∼32–55% for BSA, ∼10–21% for OVA) were substantially higher than those for the other identified amino acids (∼5–12% for BSA, ∼4–6% for OVA). Upon oxidation of tripeptides with Gly in C-terminal, mid-chain, or N-terminal positions, Gly was preferentially released when it was located at the C-terminal site. Overall, we observe evidence for a site-selective formation of free amino acids in the OH radical-induced oxidation of peptides and proteins, which may be due to a reaction pathway involving nitrogen-centered radicals.

**Electronic supplementary material:**

The online version of this article (doi:10.1007/s00216-017-0188-y) contains supplementary material, which is available to authorized users.

## Introduction

Reactive oxygen species (ROS) have been associated with various diseases (e.g., diabetes and cancer), as they can cause oxidative stress, biological aging, and cell death [1–7]. The hydroxyl radical (OH), the most reactive form of ROS, can oxidize most organic compounds such as proteins and DNA [8]. Hydroxyl radicals can be generated in biological systems endogenously and exogenously [9], and the sources include a variety of different processes such as cellular metabolic processes, radiolysis, photolysis, and Fenton chemistry [10–12]. Elucidation of the OH-induced oxidation mechanism of amino acids, peptides, and proteins is of exceptional importance for physiological chemistry (e.g., for understanding the relationship between protein oxidation and aging) [13–16] and also of considerable interest for the Earth’s atmosphere [17, 18].

Hydroxyl radicals undergo several types of reactions with amino acids, peptides, and proteins. Typical reactions include addition, electron transfer, and hydrogen abstraction [14, 15]. The OH radicals can attack both amino acid side chains and the peptide backbone, generating a large number of different radical derivatives of proteins [19, 20]. With respect to the peptide backbone cleavage, the main reaction pathway is initiated by an H abstraction at the α-carbon position. This is followed by a reaction with O_2_ to give a peroxyl radical, which ultimately results in fragmentation and cleavage of the backbone of the protein, thereby mainly forming amide and carbonyl fragments [11, 21]. Several studies have demonstrated that the H abstraction from the α-carbon position is the dominant pathway for the OH-mediated fragmentation of proteins and occurs at specific sites or amino acid residues as shown by computational and experimental investigations [9, 22, 23]. Also, the metal-catalyzed oxidation (MCO) of proteins was found to be an important pathway for protein degradation, as metal ions preferentially bind particular sites of proteins, resulting in selective damage [14, 24–26]. Among the multiple oxidation products, carbonyl compounds, peptide-bound hydroperoxides, and larger protein fragments were predominantly identified [27–30]. For example, Morgan et al. [28] investigated the site selectivity of peptide-bound hydroperoxide and alcohol group formation, as well as fragment species formed through protein oxidation by OH/O_2_ using a mass spectrometry (MS) approach.

The high reactivity of proteins with OH radicals, however, may result in various products due to different reaction mechanisms [31, 32]. In this study, we focus on the identification and quantification of amino acids as oxidation products of proteins and peptides generated by hydroxyl radicals from the Fenton reaction. For this purpose, we introduced two robust analytical methods based on mass spectrometry and liquid chromatography, which have been widely used for the determination of amino acids in various environments (e.g., plasma and plant extracts) [33, 34]. These methods provide analytical evidence for the release of amino acids due to the OH-mediated oxidation of peptides and enable their yields to be quantified.

Bovine serum albumin (BSA) and ovalbumin (OVA) were used as model proteins, and tripeptides with varying amino acid sequences were used to study yields and site selectivity for reactions with OH radicals. The amino acids consisted of the tripeptides (glycine (Gly), alanine (Ala), serine (Ser), and methionine (Met)) were chosen due to their reactivity towards OH radicals; i.e., Gly, Ala, and Ser show a low reactivity towards OH, while the rate constant of Met with OH is about 2 orders of magnitude higher [19]. Oxidation products were analyzed by high-performance liquid chromatography tandem mass spectrometry (HPLC-MS/MS) using a Q-ToF mass spectrometer and pre-column online *ortho*-phthalaldehyde (OPA) derivatization-based amino acid analysis by HPLC with diode array detection and fluorescence detection to identify and quantify free amino acids. We report the release of free amino acids in the OH radical-induced oxidation of peptides and proteins. Furthermore, effects of amino acid side chains on the release are discussed with regard to product identification and site selectivity.

## Experimental

### Reagents

BSA (A5611), OVA (grade V, A5503), Gly-Gly-Gly ((Gly)_3_, G1377), Met-Ala-Ser (M1004), NaH_2_PO_4_·H_2_O (71504), OPA (P0657), 9-fluorenylmethoxycarbonyl chloride (FMOC-Cl, 23186), 3-mercatopropionic acid (63768), acetonitrile (ACN, 34998), methanol (MeOH, 494291), amino acid standards (AAS18), asparagine (A0884), glutamine (49419), tryptophan (93659), sodium tetraborate decahydrate (Na_2_B_4_O_7_·10H_2_O, S9640), FeSO_4_·7H_2_O (F7002), H_2_O_2_ solution (30%, *w*/*v*, 16911), and HCl solution (0.1 M, 318965) were purchased from Sigma-Aldrich (Germany). Sodium hydroxide (NaOH, 0583) was from VWR (Germany). Met-Gly-Ala, Gly-Ala-Met, and Ala-Met-Gly were obtained from GeneCust (Luxembourg) and were delivered in the desalted form with a purity >95%. High purity water (18.2 MΩ cm) was taken from an ELGA LabWater system (PURELAB Ultra, ELGA, UK) and autoclaved before use if not specified otherwise.

### Protein/peptide oxidation reactions

**Fig. 1:**
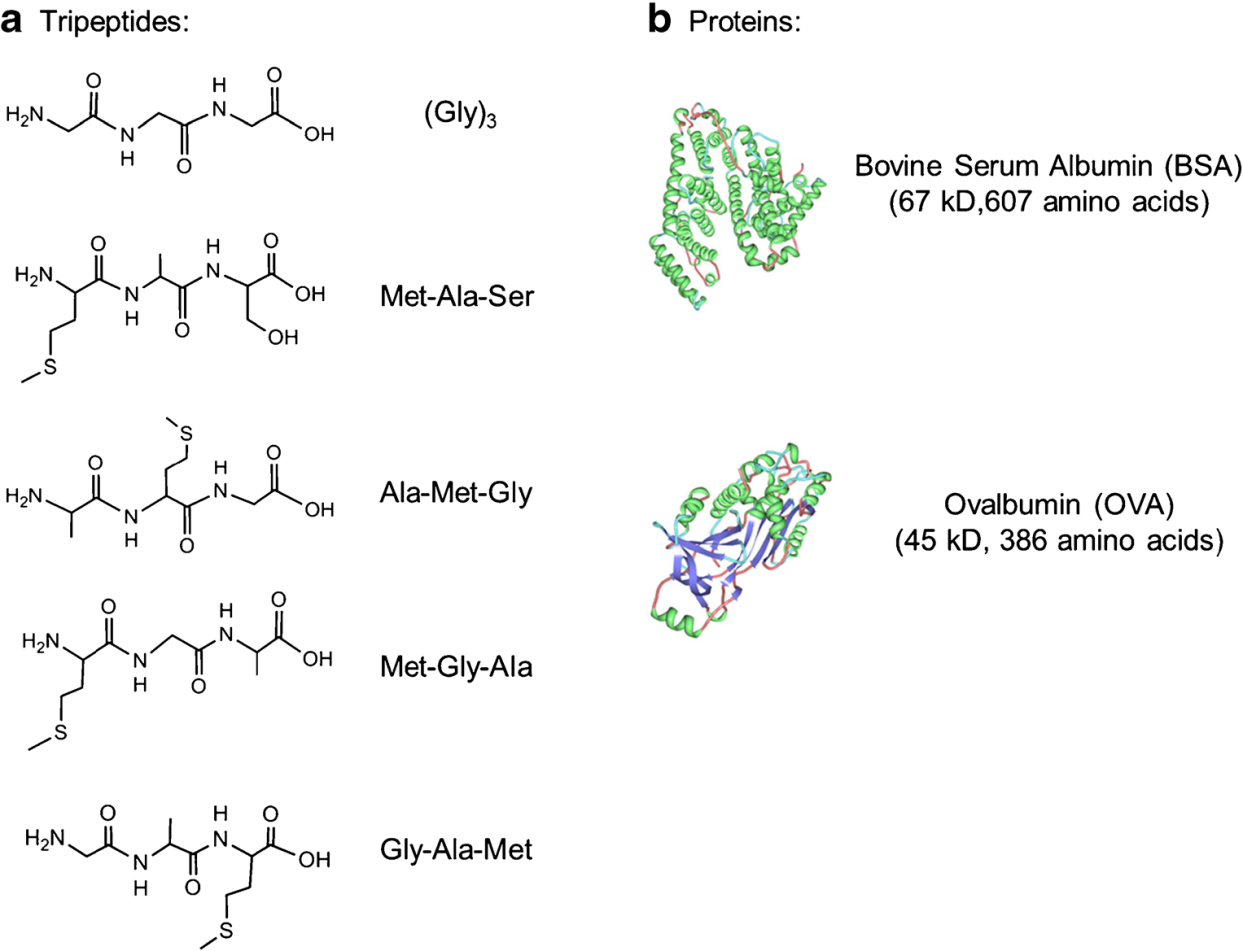
Structures of the investigated peptides (**a**) and proteins (**b**) in this study. The molecular structures of proteins (BSA, PDB accession number 3V03; OVA, PDB accession number 1OVA) were created using the RCSB PDB protein workshop (4.2.0) software

Reaction mixtures of proteins/peptides (structures shown in Fig. 1) with Fenton oxidants (FeSO_4_-H_2_O_2_) were stirred (Multistirrer 15, Fischer Scientific, Germany) in closed screw-cap vials at room temperature. Hydroxyl radicals were generated under two oxidation conditions, and the estimated effective OH concentrations are listed in Table 1. The pH of the reaction solutions was adjusted to 3 by adding 1 M NaOH and measured by a pH meter (Multi 350i; WTW, Weilheim, Germany). Although ethylenediaminetetraacetic acid (EDTA) is a common chelator to stimulate the generation of radicals under physiological pH conditions (pH 6–8) [35], no EDTA was added in this study as glycine was found to be one of the degradation products of EDTA in the presence of OH [36]. For protein oxidation reactions, the proteins BSA and OVA were pretreated with a size-exclusion column (PD-10, GE Healthcare, Germany) using ultrapure H_2_O to remove low molecular components (<5 kDa). From the purified 25 mg mL^−1^ protein solutions, 100-μL aliquots were added to the Fenton oxidant solutions to a final volume of 2.5 mL. After the respective reaction times, the oxidized samples were immediately eluted on a PD-10 column pre-equilibrated with ultrapure H_2_O to separate the protein and the low molecular weight fraction (<5 kDa). For peptide oxidation reactions, 100 μL of 100 mM solutions of the investigated peptides were added as described before. Control reactions were performed using either H_2_O_2_ or FeSO_4_ alone at the same concentrations and pH conditions, adjusted by 0.1 M HCl and 1 M NaOH, respectively.

**Table 1:** Oxidation conditions for the generation of OH radical in aqueous solutions

Condition	Compositions		pH (adjusted by 1 M NaOH)	[OH] (molecule cm^−3^)^a^
	FeSO_4_ (mM)	H_2_O_2_ (mM)		
Ox1	5	50	3	1.5 × 10^8^
Ox2	5	150	3	2.1 × 10^8^

^a^The decay of (Gly)_3_ was monitored and allowed for an estimation of the effective OH concentration based on a pseudo-first-order kinetic rate function: [(Gly)_3_] = [(Gly)_3_]_0_ *e* ^(−*k*[OH]*t*)^, where [(Gly)_3_] is the recovery of (Gly)_3_, [(Gly)_3_]_0_ is the initial recovery (i.e., 100%), *k* (1.2 × 10^−12^ cm^3^ s^−1^) is the second-order rate constant for the reaction of OH with (Gly)_3_ [16], [OH] is the effective concentration of hydroxyl radical (assuming it remains constant during the reaction), and *t* is the reaction time. The fitting curves are shown in Fig. S6 in ESM

In addition, oxidation experiments were performed for peptides with UV-induced OH generation via the homolysis of H_2_O_2_ in aqueous solution. Briefly, 4 mM (Gly)_3_ were mixed with 50 mM H_2_O_2_ or 200 mM H_2_O_2_ in a 10 × 10 × 40 mm UV quartz cuvette (Hellma Analytics, Müllheim, Germany) and subsequently irradiated by four UV lamps (wavelength of 254 nm, LightTech, Hungary) for 1 h. The pH of these samples was also adjusted to 3 by adding 0.1 M HCl. Control samples were either treated the same way as described above, but without UV irradiation, or prepared without H_2_O_2_ and irradiated for 1 h.

All experiments described above were performed in duplicate, and the samples were lyophilized (−40 °C, ∼12 h) immediately after reaction to stop the reaction by removing the hydrogen peroxide. The dry residues were stored at −20 °C and redissolved in 100 μL H_2_O for analysis.

### Amino acid analysis

The oxidized peptides and low molecular weight fraction of proteins were analyzed with the HPLC-DAD-FLD system (Agilent Technologies 1200 Series) consisting of a binary pump (G1312B), a four-channel microvacuum degasser (G1379B), a column thermostat (G1316B), an autosampler with a thermostat (G1330B), a photo-diode array detector (DAD, G1315C), and a fluorescence detector (FLD, G1321A). ChemStation software (version B.03.01, Agilent) was used to control the system and for the data analysis.

Chromatographic conditions were in accordance with the instructions by Agilent Technologies [37]. Briefly, automatic pre-column derivatization with OPA and FMOC was performed at room temperature, according to the injector programs (for details, see Table S1 in Electronic Supplemental Material (ESM)) listed in Henderson et al. [37]. After derivatization, an amount equivalent to 0.5 μL of each sample was injected on a Zorbax Eclipse amino acid analysis (AAA) column (150 mm × 4.6 mm i.d., 3.5 μm, Agilent) at a temperature of 40 °C. Mobile phase A was 40 mM NaH_2_PO_4_ (aq), adjusted to pH 7.8 with 10 N NaOH (aq), while mobile phase B was acetonitrile/methanol/water (45:45:10, *v*/*v*/*v*). The flow rate was 2 mL min^−1^ with a gradient program that started with 0% B for 1.9 min followed by a 16.2-min step that raised eluent B to 57%. Then, eluent B was increased to 100% within 0.5 min and kept for another 3.7 min. The mobile phase composition was reset to initial conditions within 0.9 min, and the column was equilibrated for 2.8 min before the next run. Primary amino acids were detected by monitoring the UV absorbance at 338 nm, with a reference at *λ* = 390 nm, bandwidth = 10 nm, slit of 4 nm, and peak width of >0.1 min, simultaneously detected by FLD with excitation 340 nm, emission 450 nm, and photomultiplier tube (PMT) gain of 10. Secondary amino acids were detected by FLD with excitation 266 nm, emission 305 nm, and PMT gain of 9. A mixture of 20-amino acid standards (see ESM Table S2) was used to obtain calibration curves for quantification as illustrated in Fig. S1 in ESM. The limits of detection (LODs, defined as a signal-to-noise ratio of 3) for 20 individual amino acids are in the range of 0.1 to 5 pmol. Linearity is demonstrated for the concentration range of 20 to 500 μM for all amino acids by detection using a DAD or FLD.

### LC-Q-TOF-MS

Identification of OH-mediated reaction products of peptides and the low molecular weight fraction of proteins was also carried out using an HPLC-MS/MS system (Agilent). The LC-MS/MS system consists of a quaternary pump (G5611A), an autosampler (G5667A) with a thermostat (G1330B), a column thermostat (G1316C), and an electrospray ionization (ESI) source interfaced to a Q-ToF mass spectrometer (6540 UHD Accurate-Mass Q-ToF, Agilent Technologies). All modules were controlled by MassHunter software (Rev. B. 06.01, Agilent). The LC column was a Zorbax Extend-C18 Rapid Resolution HT (2.1 × 50 mm, 1.8 μm) and was operated at a temperature of 30 °C. Eluents used were 3% (*v*/*v*) acetonitrile (Chromasolv, Sigma, Seelze, Germany) in water/formic acid (0.1% *v*/*v*, Chromasolv, Sigma, Seelze, Germany) (eluent A) and 3% water in acetonitrile (eluent B). The flow rate was 0.2 mL min^−1^ with a gradient program starting with 3% B for 1.5 min followed by an 18-min step that raised eluent B to 60%. Further, eluent B was increased to 80% at 20 min and returned to initial conditions within 0.1 min, followed by column re-equilibration for 9.9 min before the next run. The sample injection volume was 1–5 μL.

The ESI-Q-TOF instrument was operated in the positive ionization mode (ESI+) with a drying gas temperature of 325 °C, 20 psig nebulizer pressure, 4000 V capillary voltage, and 75 V fragmentor voltage. Fragmentation of protonated ions was conducted using the targeted MS/MS mode with a collision energy of 10 V (16 V for *m*/*z* 76). Spectra were recorded over the mass range of *m*/*z* 50–1000 for MS mode and *m*/*z* 20–1000 for MS/MS mode. Data analysis was performed using the qualitative data analysis software (Rev. B. 06.00, Agilent).

## Results and discussion

### Identification of amino acid products in the hydroxyl radical-induced oxidation of peptides and proteins

Figure 1 shows the tripeptides and proteins investigated in this study. The oxidation products generated by OH radicals from the Fenton reaction were analyzed by AAA and LC-MS/MS in order to identify and quantify amino compounds and, in particular, amino acid products.

**Fig. 2:**
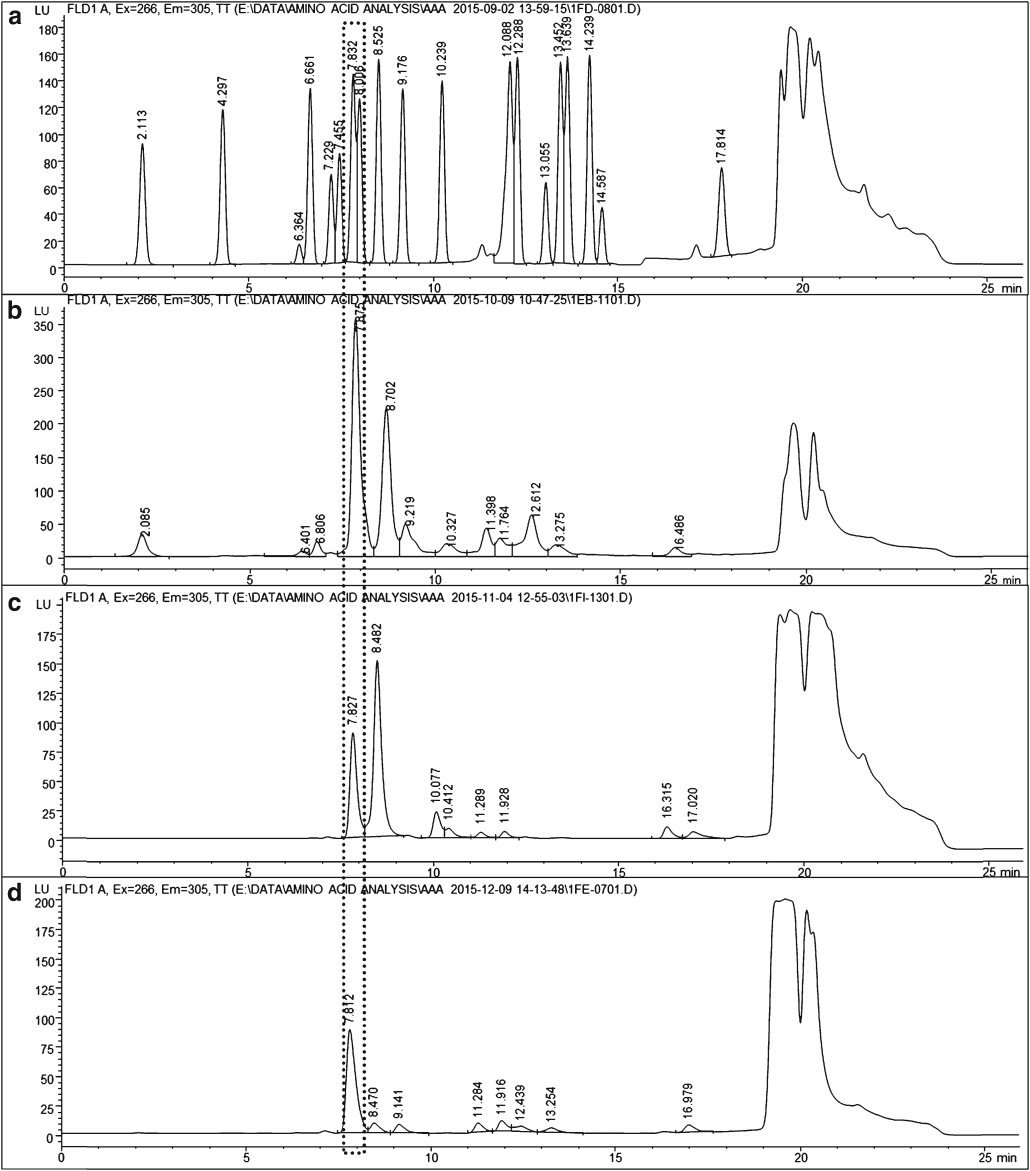
Amino acid analysis (AAA) with fluorescence detection of OPA-derivatized amino acids: (**A**) 200 μM of a 20-amino acid standard; (**B**) 15 μM BSA, Ox2, 24 h; (**C**) 4 mM tri-Gly, Ox1, 0.25 h; and (**D**) 4 mM Ala-Met-Gly, Ox1, 19 h. The *dotted box* indicates the signal of glycine in all samples

**Fig. 3:**
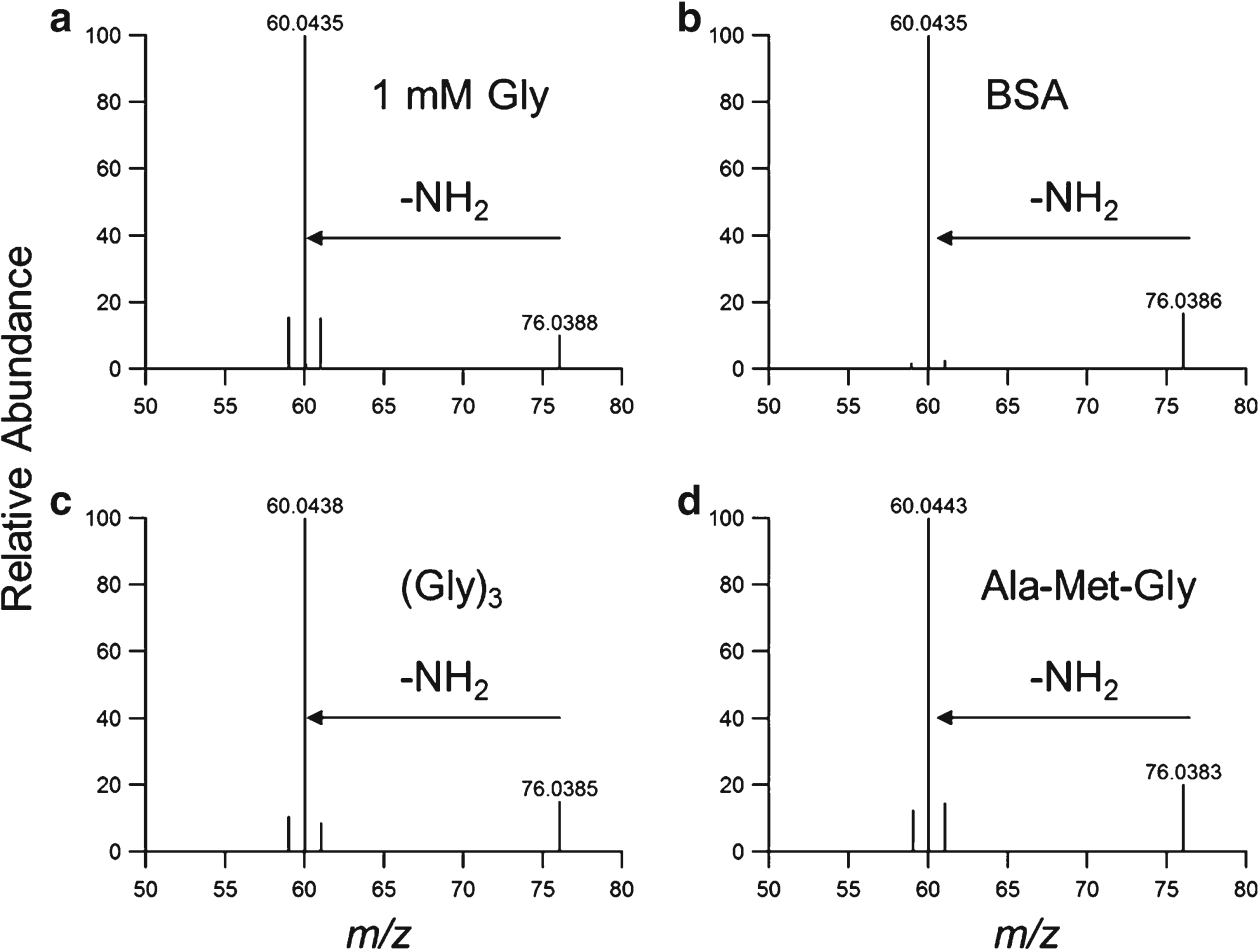
The MS^2^ spectra of *m*/*z* 76 in (**A**) 1 mM Gly, (**B**) oxidized BSA sample in Ox2 condition, (**C**) (Gly)_3_ in Ox1 condition, and (**D**) Ala-Met-Gly in Ox1 condition (Ox1, 5 mM FeSO_4_–50 mM H_2_O_2_; Ox2, 5 mM FeSO_4_–150 mM H_2_O_2_). The oxidized samples show an accurate mass of precursor ion *m*/*z* 76 with the glycine standard, and they exhibit the same fragments of *m*/*z* 60. The extracted ion chromatograms (EICs) of *m*/*z* 76 for the above samples are shown in Fig. S2 in ESM

Figure 2 shows the exemplary AAA chromatograms of an amino acid standard, as well as protein and peptide samples oxidized by OH radicals. The signal corresponding to glycine-OPA derivative at a retention time (RT) of 7.8 min was detected in all oxidized samples of glycine-containing peptides and proteins. Moreover, the peak was absent when the oxidized peptide did not contain glycine (i.e., Met-Ala-Ser). LC-MS/MS analysis of underivatized samples further confirmed the free amino acid glycine to be an oxidation product of proteins and peptides reacting with hydroxyl radicals. Figure 3 shows the MS/MS spectra of a glycine standard (*m*/*z* 76) and those of precursor ions with *m*/*z* 76 found in oxidized BSA, (Gly)_3_, and Ala-Met-Gly samples. In all cases, identical fragmentation patterns were observed and the loss of 16 Da from the precursor ions corresponds to the loss of NH_2_ [34]. In addition, the signal intensity of extracted ion chromatograms (EICs) for *m*/*z* 76 in the oxidized samples increased significantly compared to the control samples (see ESM Fig. S2), indicating the formation of an OH-mediated reaction product with *m*/*z* 76 in these samples. Thus, glycine, which does not contain an oxidation sensitive side chain, could be identified as a product of all studied reaction systems of peptides and proteins comprising glycine in their amino acid sequences.

In addition to glycine, three other peaks exhibiting the RT of OPA derivatives of aspartic acid (Asp), asparagine (Asn), and Ala were detected in the AAA of oxidized protein (BSA and OVA) samples, i.e., at 2.1 min for Asp, 6.4 min for Asn, and 9.2 min for Ala, as illustrated in Fig. 2B. The LC-MS/MS analysis of reference compounds and samples confirmed the identity of the amino acids as shown in Fig. S3 in ESM [34, 38]. It should be noted that the four free amino acids (Asp, Asn, Gly, and Ala) identified in oxidized protein samples, all exhibit a low rate constant for reactions with OH [19, 39], resulting in a higher stability towards further reactions with OH radicals and enabling their identification in the analysis. Furthermore, Ala and Asp were unambiguously identified by LC-MS/MS in the oxidized Met-Gly-Ala and Gly-Ala-Met samples. Exemplary MS^2^ spectra of reference standards and samples are shown in Fig. S4 in ESM. The presence of Asp in the tripeptide samples can be explained by the OH-induced oxidative modification of methionine (Met), as suggested by Xu and Chance [11] and illustrated in Fig. S5 in ESM. Note that Asp was not identified in the oxidized Ala-Met-Gly sample. This discrepancy may be explained by the formation of other oxidation products of Met, which can be formed when Met is located in the middle of the peptide, as Met is highly reactive towards OH and the reaction could result in different oxidized species [11]. In the oxidized Met-Ala-Ser sample, the amino acids Asp, Ala, and Ser were identified. Here, Ser could be released directly from the C-terminal position or it could be formed by the oxidation of the methyl side chain of Ala released from the peptide [40]. Therefore, from the combined AAA and LC-MS/MS results, we can confirm that free amino acids are products in the OH-induced oxidation of proteins and peptides.

### Quantification and site selectivity of amino acid formation

**Fig. 4:**
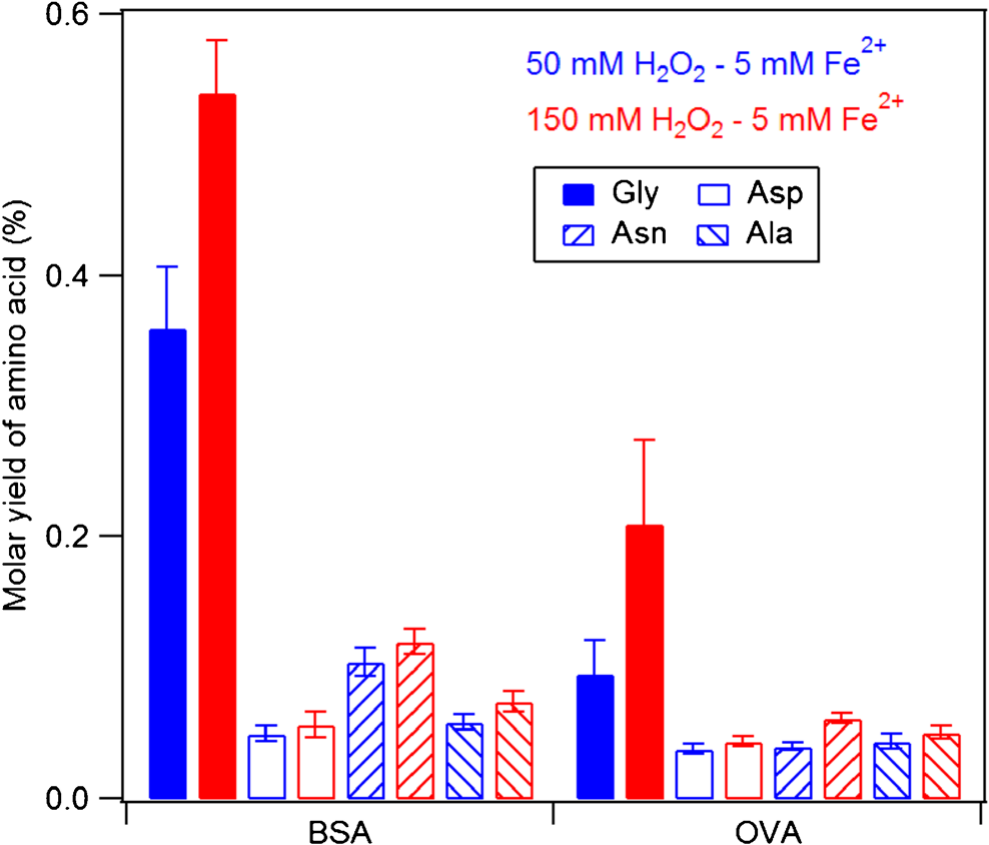
Molar yields of amino acids obtained in the oxidation of BSA and OVA samples with different concentrations of oxidants (50 and 150 mM H_2_O_2_ with 5 mM FeSO_4_, respectively)

Figure 4 shows the molar yields of free amino acids for the OH oxidation of two model proteins (BSA and OVA) quantified by AAA, whereby yields increased with increasing oxidant concentrations. The yields of Gly were found to be the highest among the quantified amino acids and ranged from ∼32 to 55% for BSA and from ∼10 to 21% for OVA. Notably, the Gly yield of BSA was approximately two to three times higher than that of OVA under the same conditions, despite the higher number of Gly residues in OVA (19) compared to BSA (17). The factors influencing the yields of individual free amino acids in the studied reactions might be multiple, including different tertiary and primary structures and thus different numbers of accessible sites available for the OH attack, as well as differences in adjacent amino acids in BSA and OVA, influencing OH site selectivity [19].

**Fig. 5:**
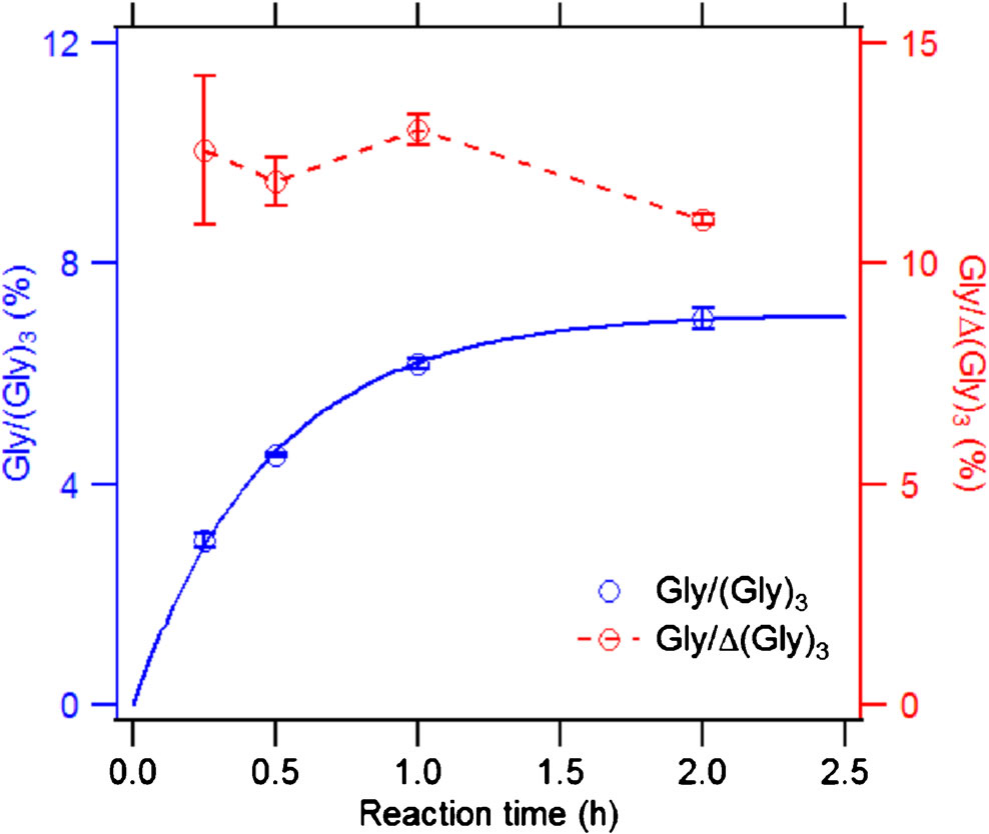
The temporal evolution of molar yield Gly/(Gly)_3_ (*blue dots*) and the product ratio of Gly to ∆(Gly)_3_ (*red dots*) in the oxidation of 4 mM (Gly)_3_ with 5 mM FeSO_4_–50 mM H_2_O_2_ condition (Ox1). ∆(Gly)_3_ was quantified by a calibration curve made by a set of (Gly)_3_ solutions (see ESM Fig. S7) monitored at a UV absorbance of 338 nm. The *solid line* (*blue*) is fitted with a pseudo-first-order kinetic rate function: [AA] = *a*[TriPep]_0_(1 − *e* ^− *k*[OH]*t*^), as discussed in the “Quantification and site selectivity of amino acid formation” section

Figure 5 shows the temporal evolution of the Gly yield during the oxidation of (Gly)_3_ by OH radicals. The corresponding recovery of (Gly)_3_ (see ESM Fig. S6) was obtained through AAA analysis using a calibration curve made by a set of (Gly)_3_ solutions (see ESM Fig. S7). We found that the recovery of (Gly)_3_ has declined to 50% after 1 h of reaction (see ESM Fig. S6), while the molar yield of glycine only reached 6% of (Gly)_3_. Additionally, the molar ratio of free Gly to reacted (Gly)_3_ (*Δ*(Gly)_3_ = (Gly)_3, *t* = 0_ − (Gly)_3, *t* = *x*_) was relatively stable over the reaction time with a value of ∼12%. These results indicate that other reaction products than Gly are accounting for ∼88% of the reacted peptide. These products may include, e.g., carbonyl species known to be products of the α-carbon H abstraction pathway [28]. To exclude an influence of acidic or basic hydrolysis on the observed formation of glycine [41], control experiments were conducted, in which (Gly)_3_ was incubated under acidic (pH 2) and basic (pH 12) conditions for 24 h, respectively. No glycine formation was observed in these experiments. Furthermore, we found that amino acids were also released in the absence of iron ions. This was confirmed through control experiment, in which OH radicals were generated by the photolysis of H_2_O_2_, and a positive relationship between glycine yield and H_2_O_2_ concentrations was observed (see ESM Fig. S8).

**Fig. 6:**
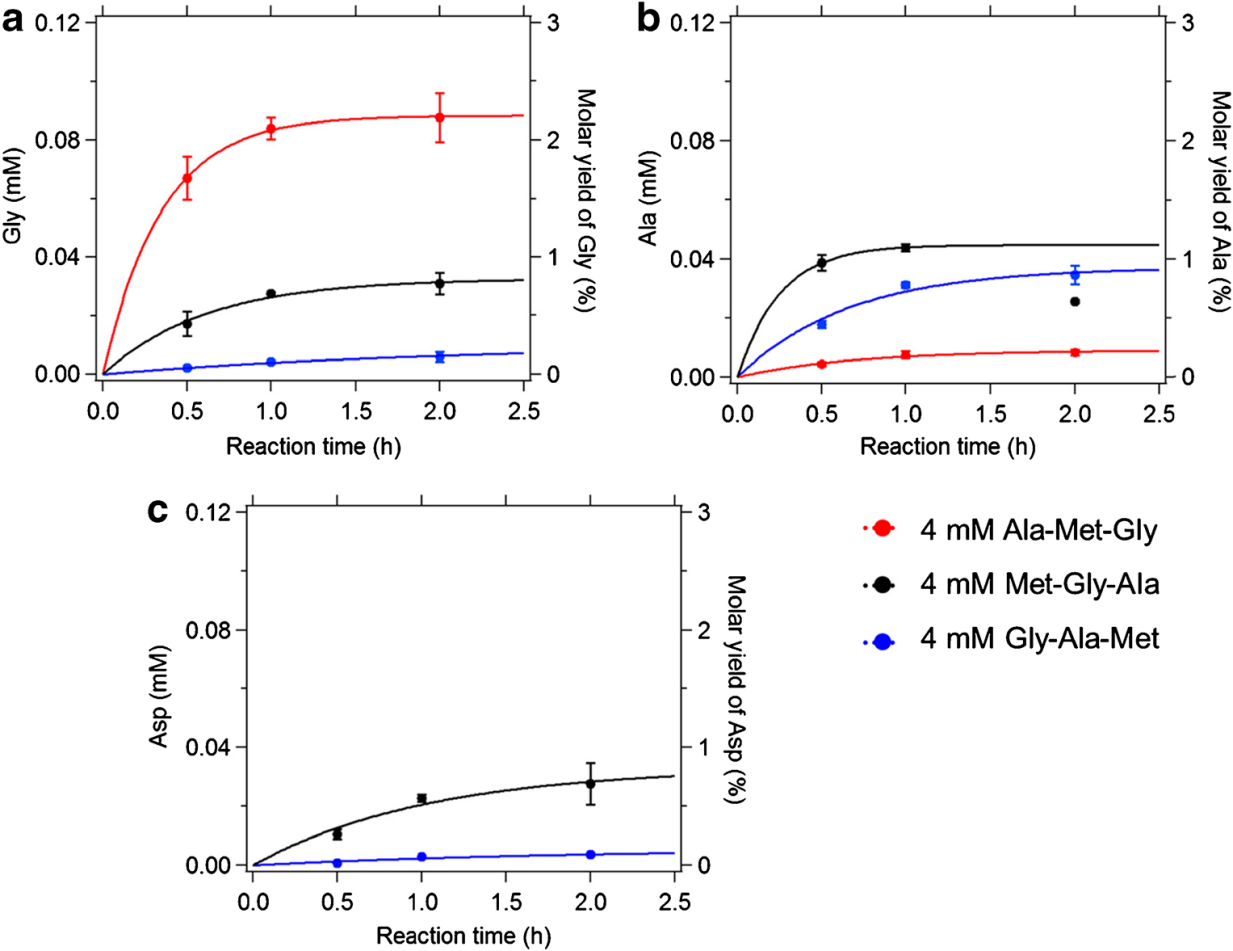
Temporal evolution of the concentration (*left axis*) and molar yield (*right axis*) of glycine (**A**), alanine (**B**), and aspartic acid (**C**) from Gly-Ala-Met, Met-Gly-Ala, and Ala-Met-Gly subjected to the oxidation with 5 mM FeSO_4_–50 mM H_2_O_2_ (Ox1). The *solid lines* are fitted with a pseudo-first-order kinetic rate function: [AA] = *a*[TriPep]_0_[1 − *e* ^− *k*[OH]*t*^], as discussed in the “Quantification and site selectivity of amino acid formation” section. For the fitting for Ala in Ala-Met-Gly, it is only fitted for the first two data points

Furthermore, we found the amino acid yields of three small peptides (Ala-Met-Gly, Met-Gly-Ala, and Gly-Ala-Met) are dependent on the sequence of Gly, Ala, and Met, as shown in Fig. 6. The highest yields of Gly and Ala were obtained when they were located at the C-terminus, followed by the mid-chain position and the N-terminal site. While the Gly concentration was increasing with reaction time, the Ala concentration already showed a reduction after 2 h of reaction time when located at the C-terminal site (Met-Gly-Ala), which may be due to further oxidation of free Ala by OH radicals. Besides, comparing the results in the case of Gly and Ala both located in the same position of the respective tripeptide, the yield of Gly was about 50% higher than that of Ala when they are located C-terminally. For mid-chain and N-terminal sites, their yields were more comparable. These results suggest that the OH attack for the release of free amino acids preferably occurs at Gly, particularly for Gly located at the C-terminal site and, to a less extent, at Ala. Previous studies have suggested that OH-mediated fragmentation of proteins likely occur at specific sites rather than giving rise to random fragments [23, 28, 42]. Glycine residues could be favorable sites for OH attacking the polypeptide backbone due to its low steric hindrance [11]. It should be noted that the highest molar yield of Gly was found to be ∼2% of the corresponding tripeptide (Ala-Met-Gly), confirming free amino acids to be low yield products and explaining the lack of reports in the literature.

Aspartic acid, the OH oxidation product of Met, was found in Met-Gly-Ala and Gly-Ala-Met. In contrast to the observed increasing yield of Gly and Ala for the C-terminal site, the Asp yields were found to be higher for the N-terminal site than for the C-terminal site, i.e., 0.7% in Met-Gly-Ala and only 0.1% in Gly-Ala-Met. The site selectivity for the OH attack at Gly may also explain why the yield of Asp was higher for Met at the N-terminal site than at the C-terminal site, since in Met-Gly-Ala, the attack on Gly may lead to the formation of Met or its oxidized product as a “byproduct”. Additionally, the temporal evolution of release for amino acids in Figs. 5 and 6 can be fitted with a pseudo-first-order rate function: [AA] = *a*[TriPep]_0_(1 − *e* ^− *k*[OH]*t*^), where the coefficient *a* stands for the maximum molar yield for the release of the specific amino acid, *k* is the second-order rate coefficient, *t* is the reaction time, and [AA], [TriPep]_0_, and [OH] are the concentrations of amino acids, tripeptide (4 mM), and OH (1.5 × 10^8^ mol cm^−3^, assuming [OH] is constant), respectively. The second-order rate coefficient for the release of amino acids from the four investigated tripeptides is in the order of magnitude of 10^−12^ cm^3^ s^−1^. The maximum molar yield for all the amino acids was from 0.0014 ± 0.0018 to 0.0709 ± 0.0011, with the highest found for Gly in (Gly)_3_ (0.0709 ± 0.0011); the detailed coefficients from fittings can be found in Table S3 in ESM. The kinetics and mechanism will be further investigated in follow-up studies.

## Conclusions

Free amino acids were identified as products in the OH-induced oxidation of proteins and peptides by LC-MS/MS analysis. In addition, the molar yields of the formation of amino acids were quantified by AAA analysis. Glycine was released at higher yields than the other identified amino acids, which is likely to be due to the absence of a side chain resulting in low rate constants for further reactions with OH and low steric hindrance of the initial radical generation on the peptide backbone, especially when Gly was in the C-terminal position. Note that the molar yields and production rates of amino acids for different peptides and proteins cannot be interchangeably used, as release of amino acids is not equal to their presence in the solution due to possible side chain oxidations of amino acids.

The formation of free amino acids, however, has not been reported for the main backbone cleavage process through α-carbon H abstraction, which results in the formation of amide and carbonyl products, as outlined in the “Introduction” section. Thus, another reaction pathway may be responsible for the formation of free amino acids. The peptide which was only composed of glycine ((Gly)_3_) appears to be a good candidate for the investigation of such pathways, because H abstraction by OH radicals can only occur at the α-carbon and the amide nitrogen. For other amino acids, however, hydroxyl radicals can attack at the side chain and polypeptide backbone sites, complicating investigations of the reaction mechanism. In previous studies, Štefanić et al. [43] determined that the amide nitrogen is the preferred site for OH attack through pulse radiolysis on free glycine and a glycine anion, whereas Doan et al. [9] concluded that H abstraction from the peptide nitrogen atom is the least preferred site for OH attack at the peptide backbone by ab initio calculations. The key difference for the contradiction in the above two studies is that the former investigated isolated amino acids while the latter used peptide systems for their calculation methods. Also, the electron transfer between sites resulting in secondary fragmentation or rearrangement [14, 44], should be considered for the formation of nitrogen-centered radicals. Further verification of the generation of nitrogen-centered radicals and the investigation of their role for the release of amino acids via protein/peptide oxidation by hydroxyl radicals could be obtained by techniques such as electron paramagnetic resonance (EPR) spectroscopy in follow-up studies [45, 46].

## Electronic supplementary material

Below is the link to the electronic supplementary material.

ESM 1 (PDF 1005 kb)
