# Supplementary material for: Release of free amino acids upon oxidation of peptides and proteins by hydroxyl radicals
Source: Anal Bioanal Chem. 2017 Jan 20;409(9):2411–20. doi: 10.1007/s00216-017-0188-y (PMC5352754; doi:10.1007/s00216-017-0188-y)
Supplement: Supplementary file 1 — (PDF 1005 kb) [file 216_2017_188_MOESM1_ESM.pdf]

Analytical and Bioanalytical Chemistry

Electronic Supplementary Material

Release of free amino acids upon oxidation of peptides and proteins by hydroxyl radicals

Fobang Liu, Senchao Lai, Haijie Tong, Pascale S. J. Lakey, Manabu Shiraiwa,
Michael G. Weller, Ulrich Pöschl, Christopher J. Kampf

Table S1 Injector program for the automatic pre-column derivatization for amino acid analysis

Step	Function	Reagent	Amount
1	draw	borate buffer	2.5 µL
2	draw	sample	0.5 µL
3	mix		3.0 µL
4	wait		0.5 min
5	draw	water (needle wash)	0 µL
6	draw	OPA-3MPA	0.5 µL
7	mix		3.5 µL
8	draw	water (needle wash)	0 µL
9	draw	FMOC	0.5 µL
10	mix		4 µL
11	draw	water	32 µL
12	mix		18 µL
13	Inject		(0.5 µL)

Table S2 A list of 20 amino acid standards used for making a calibration curve for amino acid analysis. "Peak No." is the amino acid elution order using Eclipse-AAA column

Peak No.	Amino acid (Abbreviation)	Molecular weight (g mol^{-1})	Retention Time (min)
1	Aspartic acid (Asp)	133.04	2.1
2	Glutamic acid (Glu)	147.05	4.3
3	Asparagine (Asn)	132.05	6.4
4	Serine (Ser)	105.04	6.7
5	Glutamine (Gln)	146.07	7.2
6	Histidine (His)	155.07	7.5
7	Glycine (Gly)	75.03	7.8
8	Threonine (Thr)	119.06	8.0
9	Arginine (Arg)	174.11	8.5
10	Alanine (Ala)	89.05	9.2
11	Tyrosine (Tyr)	181.07	10.2
12	Cystine (Cy2)	240.02	11.5*
13	Valine (Val)	117.08	12.1
14	Methionine (Met)	149.05	12.3
15	Tryptophan (Trp)	204.09	13.1
16	Phenylalanine (Phe)	165.08	13.5
17	Isoleucine (Ile)	131.09	13.6
18	Leucine (Leu)	131.09	14.2
19	Lysine (Lys)	146.10	14.6
20	Proline (Pro)	115.06	17.8

* Cy2 does not fluoresce under these derivatization conditions and thereby the retention time is monitored in DAD signal (338 nm).

Table S3 The fitting coefficients with a pseudo-first order rate equation: $[AA] = a[TriPep]_0(1 - e^{-k[OH]t})$, for the temporal evolution of release of amino acids in the four investigated tripeptides

Tripeptide	a			k (cm^3 s^{-1})			Chisquare		
	Gly	Ala	Asp	Gly	Ala	Asp	Gly	Ala	Asp
$(Gly)_3$	0.0709 ± 0.0011			(3.87 ± 0.28) × 10^{-12}			2.05× 10^{-6}		
Ala-Met-Gly	0.0221 ± 0.0001	0.0023 ± 0.0002		(5.32 ± 0.21) × 10^{-12}	(2.69 ± 1.06) × 10^{-12}		4.13× 10^{-7}	4.06× 10^{-7}	
Met-Gly-Ala	0.0082 ± 0.0006	0.0112	0.0083 ± 0.0022	(3.02 ± 0.88) × 10^{-12}	7.36 × 10^{-12}	(1.77 ± 1.33) × 10^{-12}	2.77× 10^{-6}	*	1.05 ×10^{-5}
Gly-Ala-Met	0.0019 ± 0.0002	0.0093 ± 0.0011	0.0014 ± 0.0018	(1.47 ± 0.40) × 10^{-12}	(2.78 ± 1.26) × 10^{-12}	(0.99 ± 2.65) × 10^{-12}	4.82× 10^{-8}	9.41× 10^{-6}	9.32 ×10^{-7}

*: Chisquare cannot be achieved due to only two datapoints were used for the fitting.

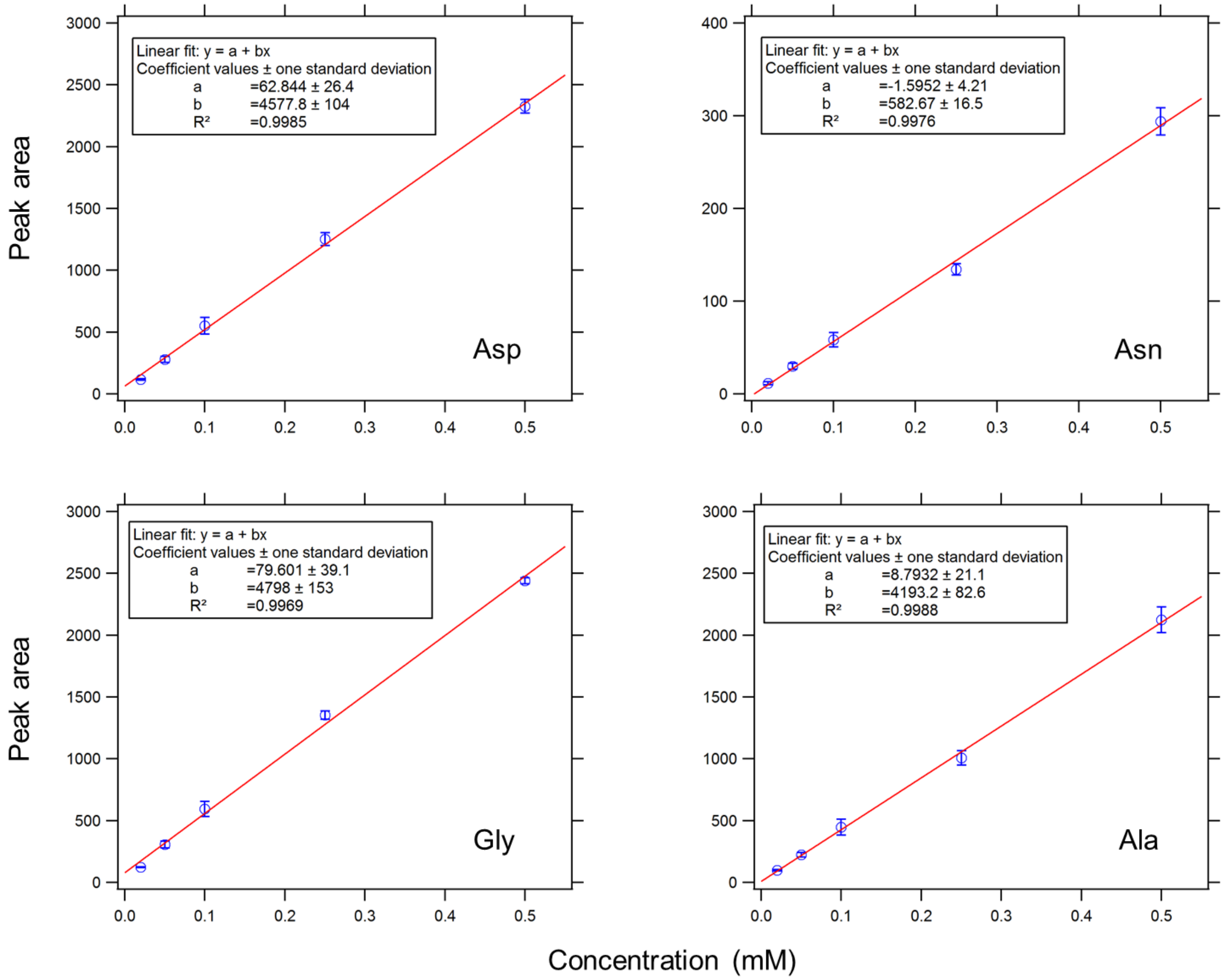

Fig. S1 Calibration curves plotting the peak area detected by FLD detection against the concentration of amino acids. (A) Aspartic acid, the fitting equation was: y = 4577.8x+62.84, R^2 = 0.999, (B) Asparagine, the fitting equation was: y = 582.67x-1.60, R^2 = 0.998, (C) Glycine, the fitting equation was: y = 4798x+79.60, R^2 = 0.999, (D) Alanine, the fitting equation was: y = 4193.2x+8.79, R^2 = 0.999

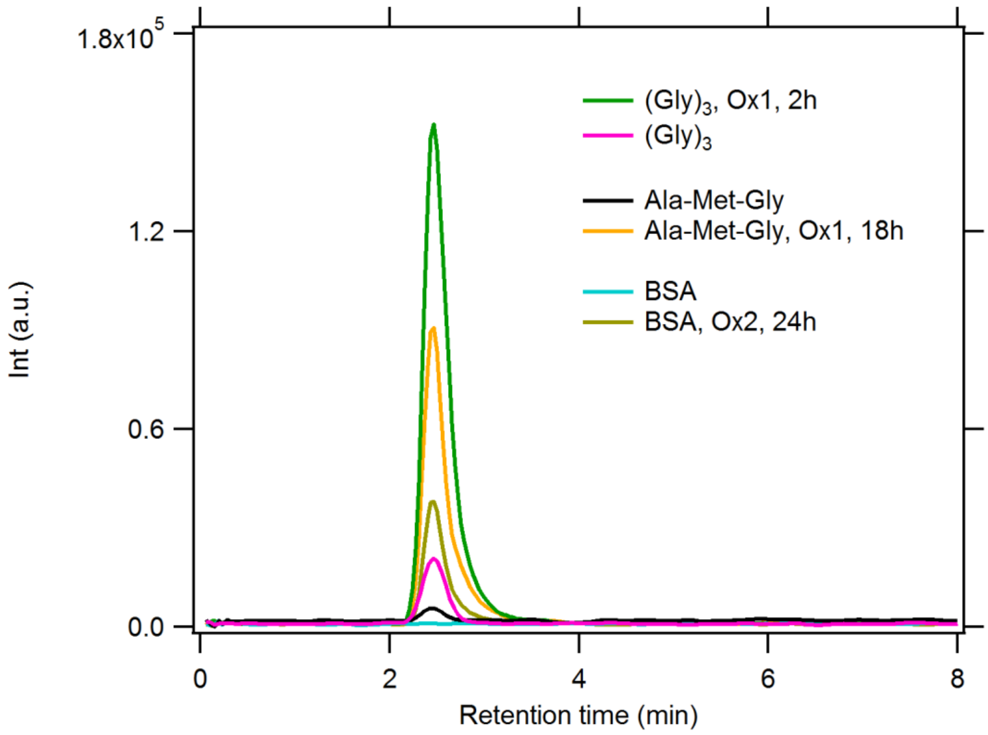

Fig. S2 The extracted ion chromatograms (EIC) of *m/z* 76 in oxidized proteins/peptides samples and respective control samples

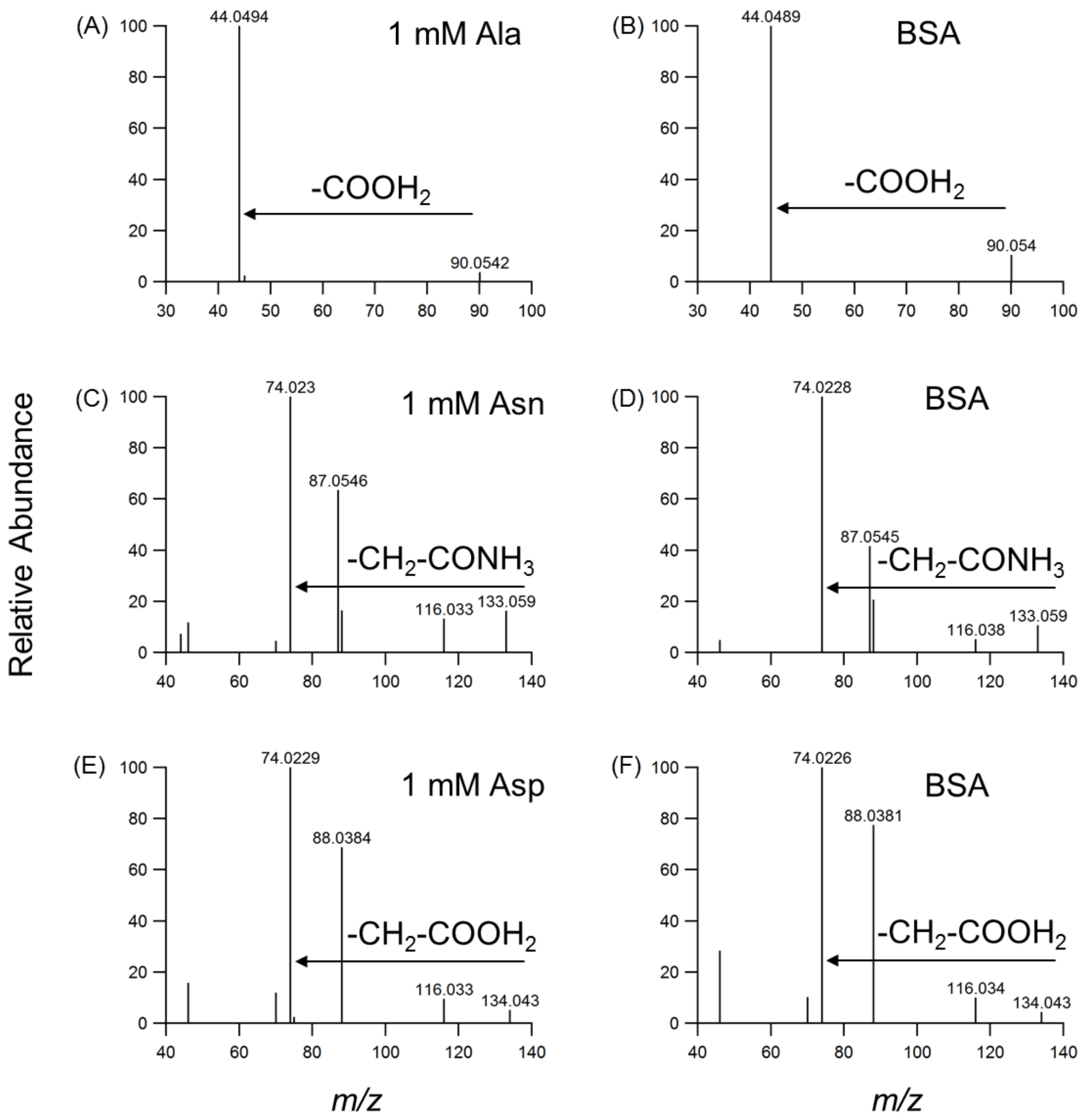

Fig. S3 The MS^2 spectra of *m/z* 90 (B), *m/z* 133 (D) and *m/z* 134 (F) in the oxidized BSA sample, in Ox2 condition (5 mM $FeSO_4$-150 mM H_2O_2).The precursor ion *m/z* 90, *m/z* 133 and *m/z* 134 was identified as alanine, asparagine and aspartic acid as they exhibited the same fragmentation patterns with *m/z* 90 in 1 mM Ala (A), *m/z* 133 in 1 mM Asn (C) and *m/z* 134 in 1 mM Asp (E), respectively

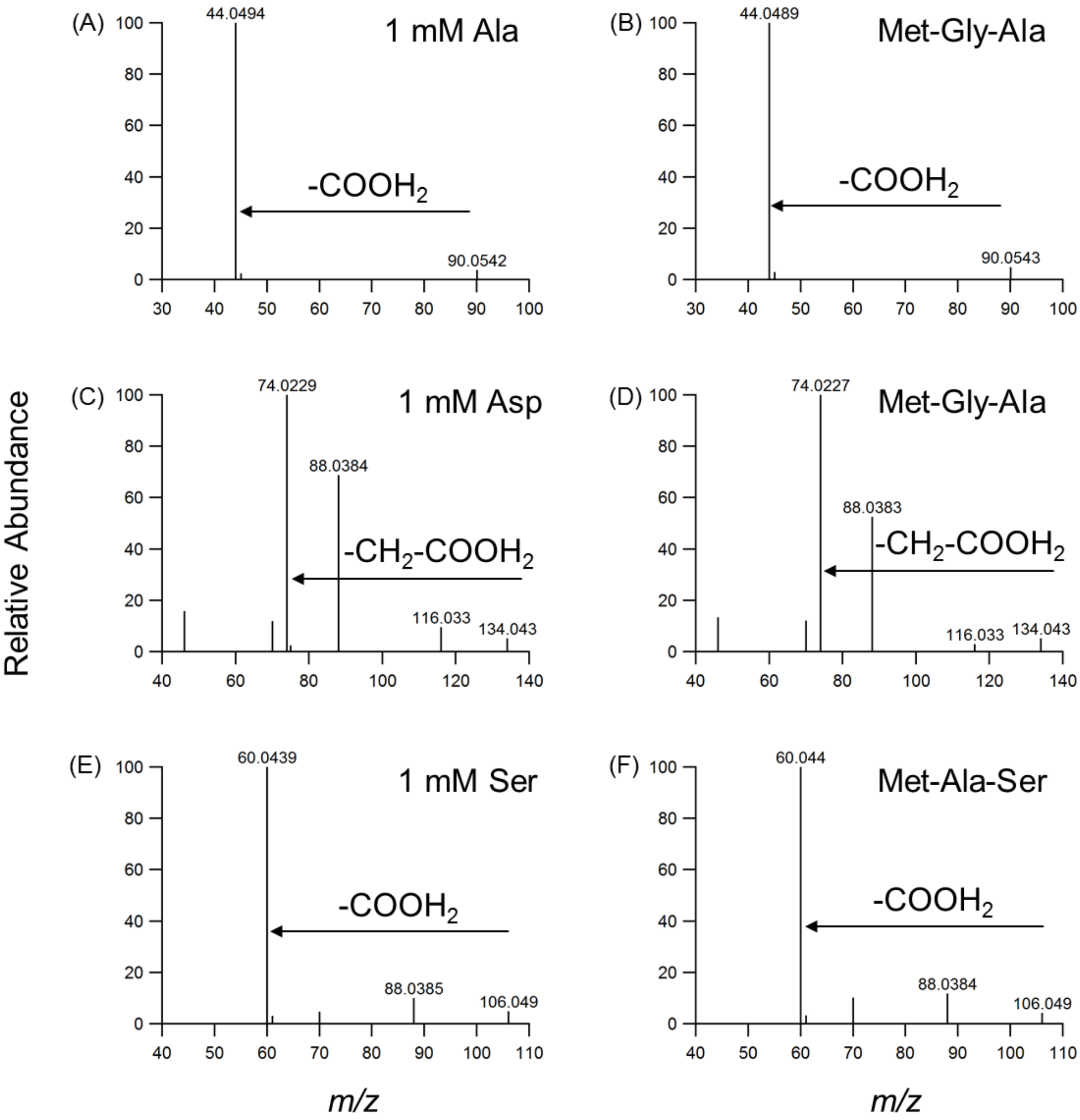

Fig. S4 The representative MS^2 spectra of *m/z* 90 (B) and *m/z* 134 (D) in the oxidized Met-Gly-Ala sample, and *m/z* 134 (F) in the oxidized Met-Ala-Ser sample in Ox1 condition (5 mM $FeSO_4$-50 mM H_2O_2).The precursor ion *m/z* 90, *m/z* 134 and *m/z* 106 was identified as alanine, aspartic acid and serine by comparison with the fragments of *m/z* 90 in 1 mM Ala (A), *m/z* 134 in 1 mM Asp (C) and *m/z* 106 in 1 mM Ser (E), respectively

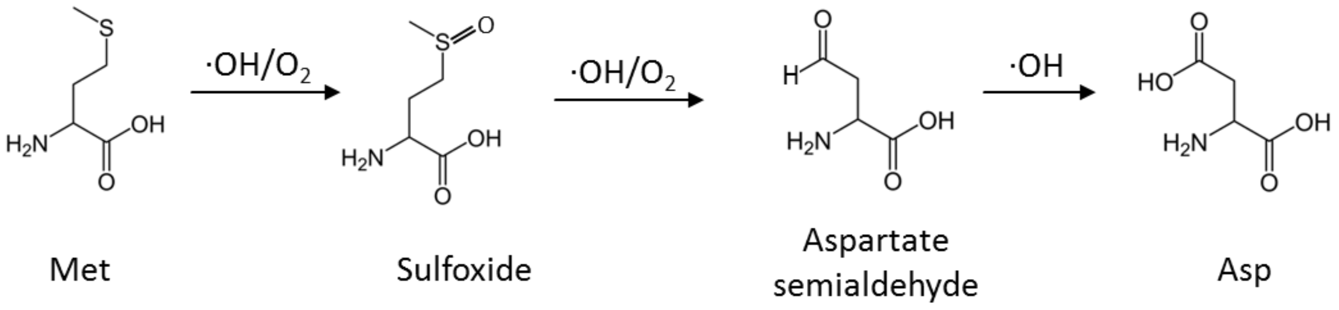

Fig. S5 Oxidation of methionine (Met) by OH radical for Met to aspartic acid (Asp) conversion. The major steps involve a first step of the oxidation of methionine to sulfoxide, followed by the formation of aldehyde at γ- carbon, which is further oxidized to yield Asp

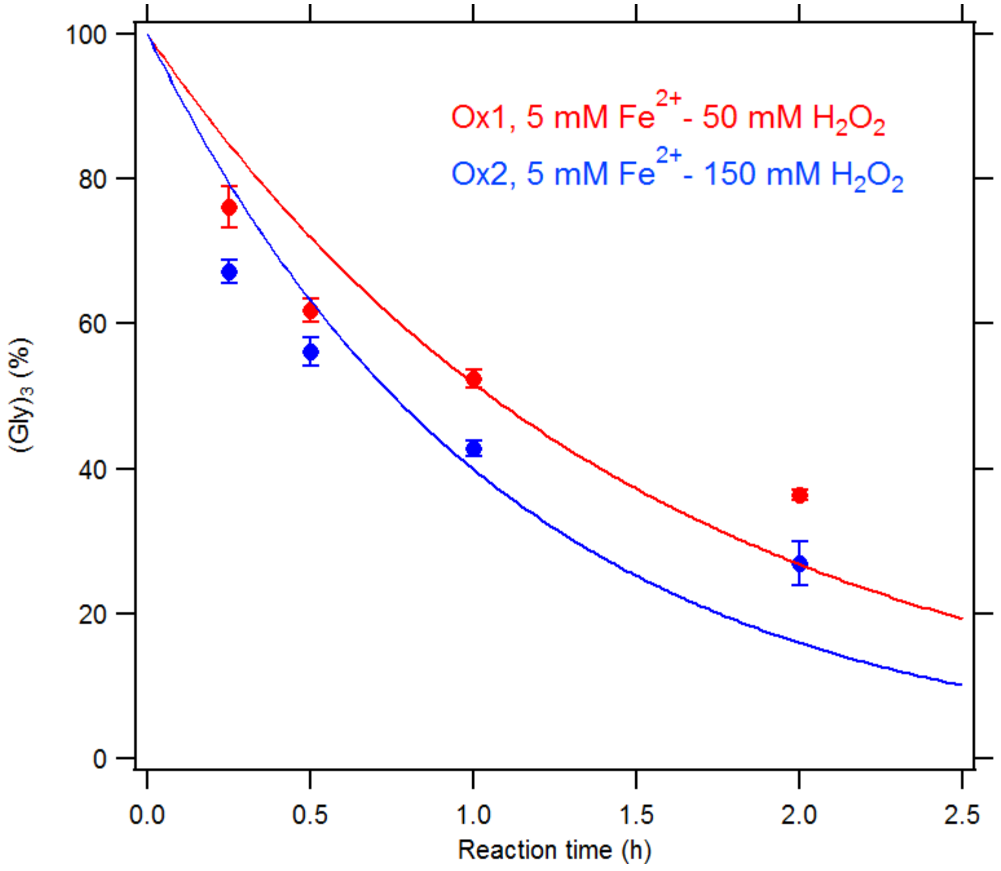

Fig.S6 The decay of $(Gly)_3$ under two different oxidation conditions. Both curves were fitted with a pseudo-first order kinetic rate function: $[(Gly)_3] = [(Gly)_3]_0 e^{(-k[OH]t)}$, where $[(Gly)_3]$ is the recovery of $(Gly)_3$, $[(Gly)_3]_0$ is the initial recovery (i.e., 100%), k (1.2×10^{-12} cm^3 s^{-1}) is the second order rate constant for the reaction of OH with $(Gly)_3$, [OH] is the concentration of hydroxyl radical, t is the reaction time. For simplification, we assumed that [OH] remained constant during the reaction in order to obtain a rough [OH] from the fitting function

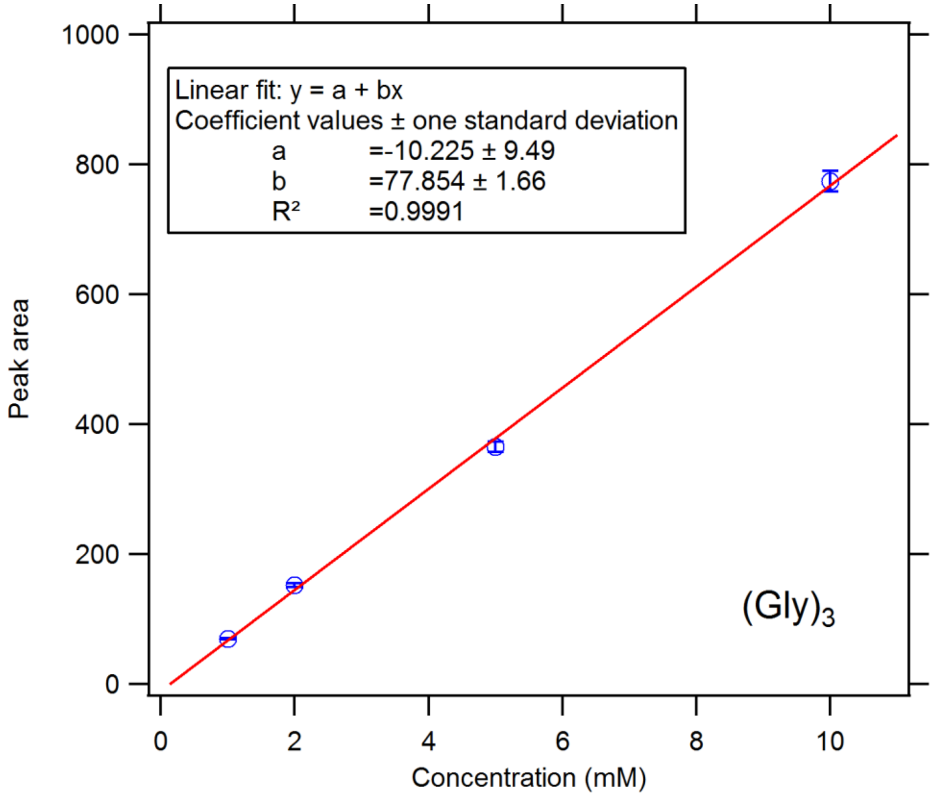

Fig. S7 Calibration curve plotting the peak area detected by DAD detection against concentration of $(Gly)_3$. The fitting equation was y = 77.85x-10.23, R^2 = 0.999

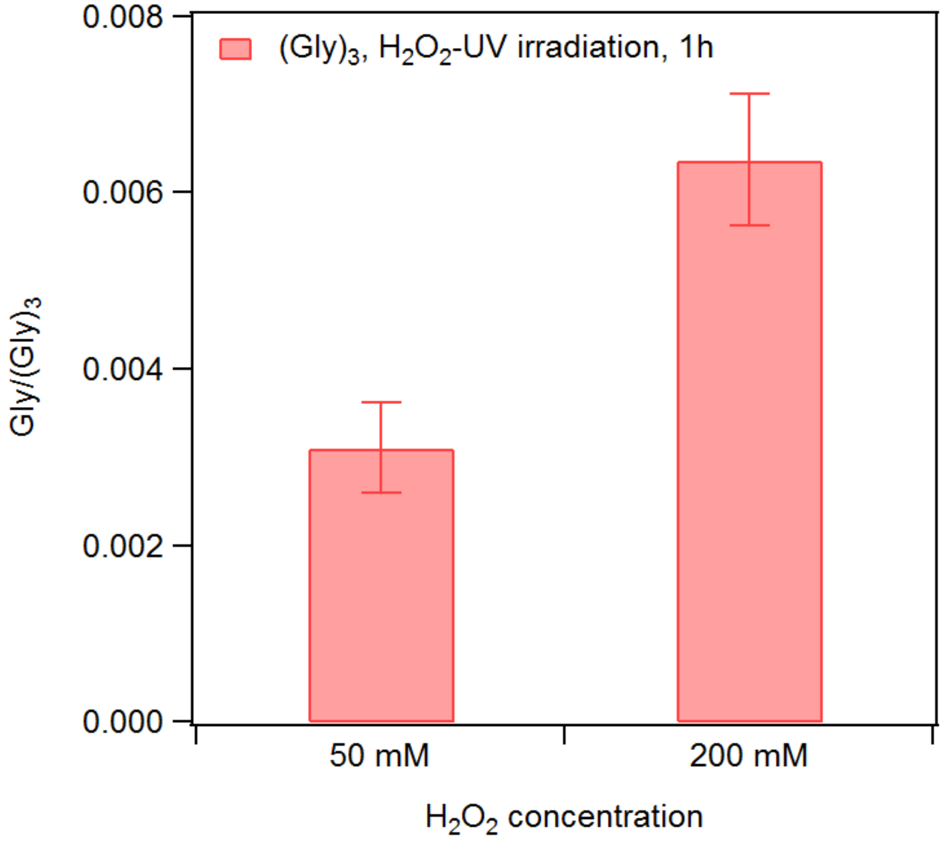

Fig. S8 Molar yield of glycine obtained in the oxidation of 4 mM $(Gly)_3$ with UV photolysis of 50 and 200 mM H_2O_2 solutions
